# Nucleic Acids Persistence—Benefits and Limitations in Forensic Genetics

**DOI:** 10.3390/genes14081643

**Published:** 2023-08-18

**Authors:** Małgorzata Żarczyńska, Piotr Żarczyński, Marcin Tomsia

**Affiliations:** 1 School of Medicine in Katowice, Medical University of Silesia, 18 Medyków Street, 40-752 Katowice, Poland; malgorzataa.zarczynskaa@gmail.com (M.Ż.); piotr.zarczynskiii@gmail.com (P.Ż.); 2 Department of Forensic Medicine and Forensic Toxicology, Medical University of Silesia, 18 Medyków Street, 40-752 Katowice, Poland

**Keywords:** acid persistence, chemical and physical factors, degradation temperature, degradation time, DNA persistence, nucleic acids degradation, post-mortem interval, radiation, RNA persistence, time since deposition

## Abstract

The analysis of genetic material may be the only way to identify an unknown person or solve a criminal case. Often, the conditions in which the genetic material was found determine the choice of the analytical method. Hence, it is extremely important to understand the influence of various factors, both external and internal, on genetic material. The review presents information on DNA and RNA persistence, depending on the chemical and physical factors affecting the genetic material integrity. One of the factors taken into account is the time elapsing to genetic material recovery. Temperature can both preserve the genetic material or lead to its rapid degradation. Radiation, aquatic environments, and various types of chemical and physical factors also affect the genetic material quality. The substances used during the forensic process, i.e., for biological trace visualization or maceration, are also discussed. Proper analysis of genetic material degradation can help determine the post-mortem interval (PMI) or time since deposition (TsD), which may play a key role in criminal cases.

## 1. Introduction

DNA has been used as confirmatory evidence in criminal investigations for a long time. DNA analysis is often the only way to convict or acquit the person of interest or identify a victim of a crime, accident, or catastrophe [1]. The introduction of highly sensitive methods of genetic material profiling broadened the extent of conditions in which nucleic acids have been assumed to persist and have enabled more efficient analyses [2]. It is a molecule consisting of two chains that wrap around each other, forming a double helix [3]. Genetic functions of DNA can be understood in two ways: the nitrogen base sequence, constituting the archive of information encoding the sequences of proteins and RNA, and the double helix structure, enabling the packaging, accessibility, and replication of the information stored in it. Most importantly, the base sequence determines both the coding of proteins, RNA molecules, and many of the physicochemical properties (i.e., stiffness, susceptibility to strand separation) [4]. Human DNA can be extracted from all nucleated cells, such as hair, tissue, blood, etc. DNA is present in 97% of home floor samples, 92% allowing for DNA profiling. In the case of cotton fabric found on the floor, DNA is present in 85% of the samples, and 79% of them enable identification [5,6]. It should be remembered that highly purified DNA is essential for molecular studies [7]. The analyses of genomic DNA (gDNA), mitochondrial DNA (mtDNA), and ancient DNA (aDNA) are currently used in many scientific fields, including forensics.

Due to the 2′ hydroxyl group of the ribose presence, RNA is less stable and more susceptible to hydrolysis than DNA. RNA is hydrolyzed in both acidic and alkaline conditions, while DNA is only hydrolyzed in acidic conditions [8,9]. After death, RNA is degraded by ribonuclease naturally occurring in the cell [10] or originating from bacteria and other environmental contaminants. The physical and non-specific chemical factors exacerbate this effect. It is well known that ribonuclease-rich organs, such as the pancreas and liver, show a rapid fragmentation of RNA [11], while other tissues, such as the brain, show much greater RNA stability [12]. DNA is also degraded by endonucleases [13] and while exposed to bacteria, fungi, or insects feeding on dead cells [14]. Nevertheless, researchers constantly attempt to find a correlation between RNA degradation and the post-mortem interval (PMI) [15]. Van den Berge et al. [16] found no relationship between the quality of mRNA sampled from the remains buried 4 to 42 years prior and suggested that the brain should be sampled for mRNA tissue profiling [16]. On the other hand, Preece et al. [17] showed that gender, age, and brain pH at death have a significant impact on the mRNA level in the human brain after death [17].

DNA and RNA of living organisms are constantly exposed to degrading factors. However, these processes are controlled due to the repair mechanisms’ presence, ensuring survival [18,19]. Repair processes stop after death, and cells and genetic material are exposed to many harmful factors, both internal and external [20]. Cell death may occur as a result of two processes: apoptosis or necrosis [21]. Post-mortem, DNA fragments are degraded by endogenous nucleases present in cells or exogenous nucleases released by microorganisms or invertebrates [9,13], especially insects. Insects are worth mentioning because, potentially, they can contaminate crime scenes or forensic laboratories [22]. Research on the third instar larvae of necrophilic *Chrysomya albiceps* showed that it is possible to obtain a complete STR profile consistent with the reference material 48 h after death [23]. Also, it is possible to isolate DNA from the insects’ feces found at a crime scene. Bacterial enzymes degrade DNA and generate a pool of small oligonucleotides of ~80–200 bp [24,25]. The external factors affecting the integrity of genetic material include light (UV), humidity, temperature, fungi, and microorganisms [26,27,28]. Despite DNA being contained in a cell, exposing tissues to the external environment will eventually physically break down the DNA strands and change its chemical structure. These changes can lead to incorrect nitrogen base pairing and the misidentification of species or individuals [29]. The most common forms of damage observed are two complementary groups of transitions: type 1 (adenine → guanine or thymine → cytosine) and type 2 (cytosine → thymine or guanine → adenine) [30]. Thus, the knowledge of the sequence of successive events is very important [29], and it is extremely important to learn about the degradation mechanisms of individual evidence materials occurring in the presence of harmful factors [31].

Below, we present the most important information related to DNA and RNA persistence. Each chapter will present interesting examples of conditions for successful and unsuccessful STR profiling. It should be remembered, however, that these are examples, not strictly defined limits of detection of the discussed method. Time is a factor that constantly influences every cell of living organisms, also after death, which enormously determines the tested genetic material’s quality. The temperature affecting nucleic acids can act as a protective or destructive factor, which is also reflected in the below-mentioned studies. Radiation, aquatic environments, and various chemical and physical factors are also mentioned. Aspects related to the substances used to visualize biological traces at a crime scene or maceration are also discussed. It is extremely important to understand the influence of various factors on the genetic material because conducting a correct DNA or RNA analysis is vital for justice system representatives when deciding on someone’s guilt or innocence.

## 2. Analysis of Genetic Material

One of the most common methods of DNA identification in forensics is the STR (short tandem repeat) polymorphism analysis [1]. It can be difficult, or sometimes even impossible, to extract DNA of high enough quality for STR typing from a low-quality sample. Sometimes, PCR amplification with commercial STR multiplexes provides partial or no information due to extensive DNA fragmentation. Many laboratories often do not undertake further analysis of these limiting samples, and some laboratories resort to single nucleotide polymorphism (SNP) analysis [32,33,34] or mitochondrial DNA sequencing of hypervariable regions [35,36].

Various highly-specific kits are used to assess the quantity and quality of sampled human DNA. They can detect genetic material as low as 32 pg in a 2 μL sample. Human genomic DNA can be detected, as well as the Y chromosome DNA itself, even when mixed with a 1000-fold greater female genetic material quantity. Commonly used kits with proven efficacy and high sensitivity include the QuantifilerTM Human DNA Quantification Kit, Quantifiler^®^ Duo DNA Quantification Kit, Quantifiler^®^ Trio Quantification Kit, Quantiplex^®^ Pro RGQ Kit, and Investigator^®^ Quantiplex HYres Kit. The above-mentioned sets allow us to accurately determine the amount of DNA in the analyzed sample, which enables the selection of the appropriate amount of template DNA for the mixture [37,38,39,40,41]. Most often, the quantitative PCR (qPCR) method or the 2100 Bioanalyzer device is used to assess the amount of RNA. The qPCR method measures the amount of the reaction product at the end of each amplification cycle or in real-time. The 2100 Bioanalyzer is a miniaturized version of agarose and acrylamide gels, which allows for analyzing just 1 µL of a sample mixed with a fluorescent dye. The undoubted advantage of this method is the ability to measure RNA integrity [42]. When working with genetic material, it is important to remember the proper decontamination of the work environment to avoid contamination of the tested samples, which may adversely affect the success of the entire analysis [43]. The most effective methods of removing cell-free DNA include sodium hypochlorite and Trigene^®^ Virkon^®^ [44].

The STR number is highly variable from person to person and at different loci [45,46]. The currently used kits enable the analysis of STR polymorphisms of non-coding autosomal DNA fragments and sex chromosomes [47]. A diploid human cell is expected to contain ~6.16 pg of DNA. Forensic research often involves the analysis of LT-DNA (low template DNA), i.e., traces characterized by low quality or quantity of DNA template, which negatively affect the results obtained. The artifacts occurring during PCR amplification result from the stochastic effects [48,49]. Most of the STR DNA kits used for DNA typing require ~100 pg of DNA [50,51]. The described method is very sensitive and, therefore, the amount of genetic material required for analysis is small, and it may be used to analyze even very old samples [52] to confirm or contradict the compliance of the analyzed samples with a high probability [53]. One of the theories assumes that in order to increase the probability of a successful analysis for a small sample, it is better to split the sample and perform two separate analyses if the amount of DNA in the sample is sufficient rather than performing one analysis, especially when analyzing LT-DNA [54]. The mini-STR method moves PCR primers as close as possible to the STR region, reducing the flanking region and, thus, the overall size of the STR marker, which in consequence, increases the success rate in obtaining a genetic profile from degraded DNA samples [55,56]. Mini-STR and SNP were used in cases where current STR kits did not allow for obtaining full DNA profiles [57,58]. The above-mentioned advantages made STR polymorphism analysis the main method of DNA identification in forensics [59].

## 3. Degradation Factors

The following sections present the selected information on factors that may affect genetic material persistence.

### 3.1. Time

#### 3.1.1. Historical Cases—Genetic Material Identification after Long Time

The DNA and RNA degradation degree depends on the quantity and type of damage accumulating over time [60,61]. It depends largely on the conditions and the integrity of the biological material when discovered [62]. Appropriate conditions allow DNA to persist for a very long time. It was possible to amplify DNA by RT-PCR from sections of the intestine of a 5000-year-old mummy found in the Tyrolean glacier [63]. Oh et al. isolated and compared the DNA of the Chinese liver fluke (*Clonorchis sinensis*) obtained from the gastrointestinal tract of the Joseon mummy and reported that the consensus sequences were 98.24 to 100% identical to the modern and ancient *C. sinensis* sequences reported from Korea, China, Japan, and other Asian countries [64]. Also, high-throughput sequencing (HTS) of mtDNA was used to obtain information on the biological sex of the 4000-year-old Djehutynakhta mummy [65]. The discovery that DNA can last hundreds of thousands of years in skeletal debris [66] and even sediment [67] greatly accelerated research on DNA analysis applications in evolutionary studies. The field of aDNA (ancient DNA) research is still expanding and exploring new areas [13]. aDNA and forensic research have a mutual requirement: the tested material must be handled carefully and approached non-invasively. DNA preserved in ancient bones, teeth, and deposits is usually very fragmented and scarce [68], and therefore, it is important to use extraction techniques with the highest efficiency factors, i.e., DNA recovery from a given template [69]. Various techniques available nowadays [69,70,71] aim at maximizing the efficiency of DNA isolation while minimizing the co-extraction of PCR inhibitors [72]. DNA adsorption on silicon dioxide particles is most widely used for aDNA purification and its concentration [73].

Until the 1980s, biological evidence was identified based on histology, microscopy, immunology, biochemistry, and serology [74]. It is worth noting that genetic material sampled in the past can be analyzed many years later and may contribute to the resolution of outdated criminal cases, like the one described by Connery [75], which had been solved nearly 30 years after the incident. Thanks to archiving previously sampled materials containing the perpetrator’s DNA, it was possible to compare it with the genetic material of others imprisoned later and indict the wanted perpetrator of a murder and sexual assault [75].

In another case, DNA sequencing helped acquit Kirk Bloodsworth in the ninth year of imprisonment following the death penalty sentence from 1984 for sexual assault and the murder of a nine-year-old girl [76,77,78]. On the other hand, others lived to experience a revoking of their initial verdict, like Charles Chatman, who spent almost 30 years in prison trying to prove his innocence. The initial verdict was based on the victim’s testimony and the blood group compliance from the crime scene. A 2002 STR analysis failed to obtain any genetic profile for the secured sperm. In 2007, a re-examination of the evidence with the new technique, Y-STR analysis, developed a sperm profile, thanks to which the initial verdict was revoked in early 2008 [77,79].

#### 3.1.2. Post-Mortem Interval (PMI) and Time since Deposition (TsD)

Since it is possible to analyze genetic material years later, it is worth knowing the correlation between PMI and DNA degradation. DNA content in the cell nucleus regularly decreases after the organism’s death, and as the PMI increases, DNA eventually disappears [80]. Williams et al. [81] sampled DNA from the spleen and brain tissues of 15 cadavers and analyzed the DNA degradation rate for 96 h at 21 °C (room temperature) and 4 °C (refrigerator). No significant DNA degradation showed in the samples stored at 21 °C and 4 °C up to 48 h. After 48 h, DNA fragmentation was more frequent in the samples stored at room temperature than in the cooled samples. Fragmented DNA was less abundant in the brain than in the spleen tissues [81]. The results confirmed earlier research reporting that the chromatin structure in brain tissue is well preserved up to 30 h after death [82]. Animal studies also confirmed that the DNA content in a rat’s retina tended to decrease with an increasing PMI [80]. Liu et al. reported that degradation of DNA from spleen lymphocyte nuclei correlates with the PMI in the first 36 h after death [83]. Research on DNA fragmentation in human blood and pig skeletal muscles showed that nuclear DNA fragmentation increases during 3–56 h after death, with most degradation taking place in 3–24 h after death [84].

Previous studies showed that RNA degradation in a dead body potentially correlates with the PMI [85,86,87]. Studies on mice showed that U6 snRNA showed the highest correlation with the PMI from day 0 to day 8 after death. U6 snRNA’s short hairpin structure and the lack of nuclease to degrade it make U6 very stable [88,89]. Studies on 18S rRNA, which is abundant in cells [90], showed that it is degraded in late PMIs and is suitable as a biomarker in cardiac and liver tissues of a dead body. β-actin may also be used as a biomarker for skeletal muscle tissue in the PMI assessment, similar to U6 snRNA [89]. Liver, heart, and skeletal muscle circRNA can also be used for a PMI determination [91]. Animal and human autopsies researching heart muscle, liver, and brain tissues showed that myocardial-specific miRNAs, such as miR-1, miR-133a, and 5S rRNA, were fairly stable for 5 or more days, even at 35 °C. miR-122 in liver tissue started to degrade after 4 days, especially at higher temperatures, and eventually, only 5S r RNA was selected to be used as a marker determining PMIs [92]. Some organs, such as the pancreas and liver, show rapid post-mortem RNA fragmentation due to a large amount of ribonuclease that activates immediately after death, while other tissues, such as the brain and heart, show significantly greater RNA stability up to 96 h after death [93,94].

Corpse decomposition is accompanied by microbiome changes as the PMI increases [95]. Thus, the analysis of microbiome genetic material can significantly help determine the time of death [96].

The time that passes since the biological trace creation (insertion), the so-called time since deposition (TsD), can also be analyzed using genetic material. An accurately estimated TsD allows not only for the witness statement or alibi verification but also for the footprint significance identification in the investigation process by linking the crime’s location and time [97].

Many different biomarkers and technologies have recently been proposed to identify body fluids and tissues of forensic importance. However, no reliable method for determining the TsD is available yet [98]. In cases of sexual assault, if the biological traces on the victim’s skin are insufficient, the effective way to secure material for DNA analysis is by performing vaginal washing of the victim. It is possible to obtain a full STR profile, even if the medical examination is delayed by about 100 h [99].

Many studies researched the possibility of determining the TsD. Bird et al. [100] studied the degradation of sperm-specific mRNA markers over 30 days. The concentrations of the investigated PRM 2 and TGM markers significantly decreased from 42.728 ng/μL and 26.465 ng/μL in the fresh sample to 13.862 ng/μL and 7.689 ng/μL one week later, respectively. Both markers continued to degrade at a similar rate for the remainder of the study. The GADPH (glyceraldehyde-3-phosphate dehydrogenase) reference gene also presented a relatively linear decline, with concentrations starting at about 7 ng/μL in a fresh sample and decreasing by approximately half each week. The researchers concluded that these different degradation rates could potentially be used to estimate the TsD of body fluids, especially for samples older than 3 weeks [100]. Bauer et al. [101] described a quantification method for RNA degradation in dried bloodstains stored for up to 15 years. It occurred that the RT-PCR-ready RNA can be isolated from blood spots regardless of their TsD, even though the RNA continues to degrade over time. The method can be used to estimate the age of the blood spots, and it detects significant differences in the RNA degradation levels between samples with at least 4–5 years of age difference [101]. Anderson et al. [102] analyzed 30 blood samples, and fresh bloodstains could be clearly distinguished from the 6-day samples in 29 of the 30 samples tested. In contrast, the 6-day bloodstains differed from the 30-day-old and older stains. Some 30-day-old stains were distinguishable from the 90-day-old ones [101]. Others could detect and extract RNA from a 23-year-old blood spot [103,104] or isolate RNA from a 16-year-old blood spot and 6-year-old saliva samples [105].

Very often, biological traces, including those for genetic material analysis, are sampled from the car related to criminal activity or accidents [106,107]. The composition and purity of samples of genetic material vary depending on where the sample is taken in the vehicle [108]. The steering wheel, seats, and dashboard usually contain the most DNA traces [109]. Interestingly, the frequency of genetic material transmission is higher in people living together due to more frequent physical contact. It is also possible to transfer DNA from outside the car to its interior [107]. It should be mentioned that the DNA obtained from the passenger seat is most often a mixture of genetic material belonging to many people [109]. Testing airbags for saliva or other biological traces’ presence seems helpful in determining the driver of the vehicle involved in the road accident [110]. However, saliva, along with genetic material, can be transferred to the airbag after its activation, also from the area surrounding the driver’s seat. Salivary α-amylase is present in 53% of the samples taken from different places in the car. In the steering wheel case, 80% of the samples were positive for saliva, with 72% belonging to the driver. However, since saliva can persist for at least 10 days in a vehicle, not every DNA profile obtained from an airbag has to match the driver in a given incident [111]. Genetic material or long-term persistence infers that DNA profiling cannot be treated as an unambiguous method for perpetrator identification [112].

Touch DNA has been an increasingly analyzed material in forensic laboratories [113]. Touch DNA persistence depends on the material that has been deposited. The latest research reports that in outdoor conditions, DNA survived the longest on fabric (9 months) and shorter on steel and rubber (6 and 3 months, respectively) [114].

In summary, DNA and RNA damage accumulates over time. Nevertheless, nucleic acids can persist for a very long time in the right conditions. Thanks to this, forensics can analyze genetic material even from ancient times; however, as the PMI increases, nucleic acid degradation progresses.

### 3.2. Temperature

Temperature plays an important role in nucleic acid degradation and persistence. Genetic material, before and after analysis, is stored in refrigerators or freezers for a long time. It has been proven that not only long-term storage of DNA samples in the freezer but also their repeated freezing and thawing have no significant effect on genetic material degradation, allowing for a re-analysis of evidence if necessary [115]. However, it should be remembered that temperature significantly changes the resistance of DNA to mechanical damage. Recent research suggests that tensile, bending, and torsion resistance decrease linearly with a temperature decrease [116].

However, as the temperature rises, the damage occurs quickly and accumulates over time [62]. Hanson and Ballantyne [117] found that high temperatures increase the magnitude and rate of change in genetic material, while lower temperatures have the opposite effect [117]. Single-molecule tethered particle motion (TPM) experiments showed that DNA’s elasticity strongly depends on temperatures in the 23–52 °C range. Research by Drissen et al. [118] showed that temperature influences not only the intrinsic properties of DNA but also the interactions with DNA-binding proteins [118]. Cossette et al. [119] studied the changes in absorbance and DNA degradation of aging bloodstains under extreme temperatures. Passive blood stains were stored in microcentrifuge tubes or on FTA cards at −20 °C, 21 °C, or 40 °C and tested at 11 timepoints over 15 days. The results showed that a higher temperature correlated with the DNA degradation rate [119]. However, the blood composition differs between individuals, which impacts bloodstains’ drying and degradation processes of [120]. Abdel Hady et al. [121] subjected blood and semen samples to various temperatures (combustion, 100 °C, 50 °C, 37 °C, 4 °C, −20 °C), and they found that a high ambient temperature affects the extracted DNA amount but less its quality. Burning, on the other hand, affects both the DNA amount and quality [121].

Abdulla et al. [122] researched the influence of temperature (55 °C, 35 °C, 25 °C, 4 °C) and humidity (41%, 55%, 58%, 62%) on the degradation of genetic material present in blood samples. At 55 °C and 41% relative humidity, the mean amount of DNA on day 1 was 40.98 ± 0.67 ng/μL. From day 2, gradual DNA degradation was observed, with no detectable DNA on day 11, indicating complete DNA degradation. Slightly gradual, but not significant, degradation was observed at 35 °C and 55% relative humidity. At 25 °C and 58% relative humidity, the amount of DNA decreased only slightly over 21 days. Finally, after 21 days at 4 °C and 68% relative humidity, no decrease or no DNA degradation was observed compared to the amount measured on day 1 [122].

Many of the new DNA persistence studies have been conducted in laboratory conditions or in temperate and subtropical climates, while little information is available on DNA persistence at tropical rainforest crime scenes like those in Singapore, as researched by Lee et al. [123]. The results of their research showed that in Singapore, where rainfall is abundant and relative humidity is high, DNA on items left outside degrades rapidly. However, when items are placed indoors, at ambient temperature, and at controlled temperature and humidity, DNA degrades, as observed in experiments conducted in temperate countries [123].

Heneghan et al. [124], in turn, researched the persistence of RNA found in bloodstains depending on the temperature (37 °C, 20 °C, 4 °C) and relative humidity (75%, 35%, 10%). The results showed that the rate of RNA degradation decreases with decreasing temperature or relative humidity. The degradation rate decreased 5–10 fold when changing the temperature from 37 °C to 20 °C or relative humidity from 75% to 35%. Likewise, the degradation rate decreased when the temperature changed from 20 °C to 4 °C or relative humidity from 35% to 10% [124].

The recovery and analysis of genetic material obtained from thermally altered human bones and teeth are gaining increasing importance in forensic research, especially in cases where no soft tissues are available for analysis [125,126]. Emery et al. [127] showed that it is possible to reconstruct entire mtDNA genomes from burnt skeletal debris exposed to temperatures < 600 °C [127].

In 2014, Hollard et al. [128] successfully obtained the STR profile, despite the high temperature affecting the analyzed material. They were able to recover a complete STR profile from the charred remains found in a Paris landfill. In this case, STR profiling was the last resort to identify the remains due to a lack of other means [128].

In summary, temperature significantly affects the nucleic acids’ stability, with low temperatures improving it; hence, the sampled material should be stored in refrigerators or freezers. Increasing temperatures speed up the degradation rate, which eventually precludes successful analyses. However, one may always try, as some studies show that promising results can be procured, even from samples stored at very high temperatures.

### 3.3. pH

Sperm detection in the material secured from a vaginal swab is often used as evidence in rape cases [129]. Vaginal pH may be key to sperm survival in the female reproductive tract and usually is around 4.5 [130]. Fonneløp et al. [129] analyzed 1223 samples that showed traces of DNA, even 72 h after the incident. In 38 samples originating from 28 cases where sperm was detected, the subsequent DNA analyses revealed that the material belonged to a crime-unrelated person (e.g., a partner), and the samples that could falsify the results were excluded. In the end, sperm was detected in 94 samples; however, DNA profiles were not obtained [129].

Traces of saliva are also a promising source of material for forensic DNA analysis and personal identification. Unfortunately, bite marks are rarely swabbed and sampled for DNA analysis. Pfeifer et al. [131] examined bite marks on apples and chocolate bars and proved that STR analysis is possible after 21 days, even in samples collected from moldy fruit. Mold reduced the amount of amplifiable DNA but did not rule out a successful STR analysis [131]. Earlier studies indicated that saliva DNA could be preserved on bite marks for 16 [132] and 24 h [133].

Dissolving bodies as a method for human remains disposal has been practiced for years. The idea behind such a crime is usually to destroy all physical evidence, obscure the cause or the time of death, or hide the victim’s identity [134]. The Metropolitan Police Forensic Science Laboratory in London conducted a series of experiments testing the effectiveness of sulfuric acid as a dissolving agent for human remains. The results showed that a skinless sheep femur dissolved within 4 days, while an amputated human foot dissolved in just 4 h. According to the researchers, the heat generated by the interaction of acid and water present in the flesh surrounding the bone was an important factor in increasing the acid’s effectiveness [135]. Vermeij et al. [135] described a case of two bodies disposed of in a barrel filled with a mixture of concentrated hydrochloric and sulfuric acid. The corpses had been dissolving for 3 weeks. After their discovery, several bone-like elements were secured, four of which had the same composition as bone (calcium and phosphorus), and were analyzed for DNA. Unfortunately, the DNA profile was obtained only for one of the victims [135].

Teeth are also good reservoirs of genetic material and allow for identifying individuals based on mitochondrial DNA extraction [134,136,137]. Hartnett et al. [138] showed that 31.45% hydrochloric acid completely digested all tissue samples, except for hair and nails, in 24 h or less, and teeth completely dissolved after 19 h [138]. Robino et al. [139] observed that DNA extracted from soft tissues completely degraded within 4 h of immersion before the tissue dissolved when testing each type of acid in an animal model [139]. On the other hand, Jadhav et al. [140] conducted a study on teeth kept in 25 mL of aqueous solutions of three different acids and periodically observed for morphological changes. The results showed that the teeth dissolved entirely in 37% hydrochloric acid after 15 h and after 20 h in 65% nitric acid. In 96% sulfuric acid, the teeth reacted differently, and even after 144 h, they observed residual sediments at the bottom of the container [140].

According to Tran and Jasra [141], 50% sodium hydroxide is able to degrade nail samples within 1 week, and the degradation is slower than in hydrochloric acid [141]. Al-Owaidi et al. [142], using STR analysis, noted a significantly decreased concentration and purity of DNA extracted from molars with hydrochloric acid and nitric acid, but to a lesser extent with sodium hydroxide [142].

Theoretically, it is possible to completely dissolve the human body without leaving any traces. The human body comprises water, fat, protein, and bone minerals, which are susceptible to acids. The only acid-resistant components in the human body are gallstones (mainly composed of cholesterol) and artificial ingredients, such as implants. For example, an 80 kg human body with 30% fat consists of approximately 40 kg of water, 24 kg of fat, 12 kg of protein, and 4 kg of bone minerals [143]. Vermeij et al. [135] state that about 8 L of 37% hydrochloric acid is required to dissolve a human body.

In conclusion, nucleic acids’ persistence also relates to the pH of the environment. A vaginal swab is a good source of material for sex offenders’ identification, despite the acidic pH of the vagina. Bite marks should be analyzed more often for DNA from saliva. Evidence completely dissolved in acids is unlikely to be suitable for DNA profiling.

### 3.4. Cleaning Agents

Body fluids are common evidence in many criminal investigations [144]. Unfortunately, such material on the clothes or bedding of the victim or the perpetrator of the crime is often washed before they are discovered and analyzed [145,146].

Visualization of semen stains on clothes washed with detergent may be difficult due to the lack of the liquid part of the semen and acid phosphatase inactivation [147]. However, visualization is possible but depends on the applied washing program and temperature, type of detergent used, and visualization method choice. A full STR analysis is usually possible, regardless of the variables mentioned above [148]. Sperm have their own chromatin structure, with less than 15% of their DNA bound to histones and the vast majority of DNA bound to protamines condensed into toroids [149], which makes the live sperm DNA better protected against degradation [150]. The detergent used during washing does not affect the integrity of the sperm cells nor damage their genetic material [151]. Noël et al. [152] showed that, in most trials, it is possible to obtain a complete genetic profile from sperm stains on fabric machine-washed up to six times. Similar results were obtained for saliva stains sampled from the material washed three times [152]. Importantly, Brayley-Morris et al. [153] showed that it is possible to obtain a full DNA profile from 8-month-old semen stains deposited on material that was then washed repeatedly, even at 60 °C with detergent. The quality of the obtained genetic material differs depending on the material [153]. It is much easier to obtain a full STR profile from cotton linen than nylon [140], most probably due to its lower absorbency and greater exposure of the analyzed material to detergents [154]. In the cases of sexual assault, it is possible to detect the victim’s vaginal mucous membrane DNA and mRNA transferred to the perpetrator’s underwear; however, such a transfer can also be demonstrated in the absence of an assault. The Bayesian analysis showed that reliable material detection depends on a high amount of DNA transferred this way [155]. Interestingly, it is also possible that DNA transfers between washed clothing items, which may lead to false accusations, for example, of sexual abuse of people from a shared household [156,157].

Visualization of blood stains after washing can also be difficult [158,159]. Kulstein and Wiegand [160] confirmed, however, that despite washing blood-stained clothes at 40 °C and 60 °C, it is possible to obtain a full STR profile from the sampled material [161]. Obtaining a high-quality DNA profile was also confirmed for fabrics washed at 90 °C by Alice et al. [152]. However, the results of Ünsal et al. [161] contradicted the results, as they reported obtaining a complete genetic profile only from fabrics washed at 40 °C and 60 °C [161].

The lack of DNA in analyzed material does not mean that no traces of RNA are present. It was confirmed that RNA, including blood-specific miRNA, was still detectable in professionally cleaned barrels of a weapon after a touchdown shot, despite the lack of DNA presence [162]. Also, mRNA markers were detectable after washing fabrics at 40 °C, while at 60 °C, such an analysis was impossible, but eventually, the amount of genetic material collected for analysis may differ depending on the washing machine and the detergents used during washing [161].

Biological traces on the victim’s body may often be the only evidence in a case [163]. William et al. [164] proved that it is possible to analyze the DNA obtained from the saliva of a victim who had showered before securing the traces [164].

Sometimes, the circumstance may require analyzing the bathing accessories. It has been shown that sperm can be obtained from a bathing sponge used by a victim [165].

The persistence of DNA originating from blood traces, saliva, and epithelial cells collected from water-rinsed, hand-washed, and dishwasher-washed knives was also checked. It was possible to obtain a full STR profile for samples collected from a tool that was water-rinsed or hand-washed with detergent, while the use of a dishwasher almost completely prevented the analysis [166].

In this section, we presented the effects of cleaning agents on genetic material. It is worth noting that there is a very high probability of a successful analysis of nucleic acids isolated from body fluid stains deposited on fabrics, even after mechanical washing. Sperm deposited on fabrics are particularly resistant to cleaning agents. However, DNA can be transferred between laundered items. The same rule applies to bathing accessories, which should be sampled for genetic material if the assault victim bathed or showered. Whenever DNA traces are absent, RNA should be analyzed.

### 3.5. Water Environment

Water greatly affects the integrity of genetic material [167]. Victims’ bodies or murder weapons are often exposed to water [168]. Water is also often chosen as a place to hide evidence [169]. That is why knowledge of DNA persistence in highly humid conditions is very important [170].

Research showed that DNA isolated from corpses stored in high-humidity environments is definitely more degraded and less useful in the analysis than DNA isolated from tissues stored in a dry environment for the same period. The difference results from a higher rate of DNA hydrolysis, which increases with the amount of water in the environment [171]. Mansour et al. [172] also confirmed that water significantly reduced the amount of DNA isolated from human teeth that could be analyzed [172]. Water also negatively affected the DNA isolated from costal cartilage [173]. Recent reports prove that the type of material on which contact DNA has been deposited in water is very important. Metal surfaces provide an environment that is more conducive to the survival of the applied cellular material compared to plastic or ceramics [174]. Schmidt et al. [175] reported that the amount of genetic material obtained from blood stains located on the shoe sole was significantly lower when shoes were used on a wet surface compared to a dry surface. A total of 99.997% of the original amount of DNA was lost after 10 steps on a wet surface, while a full STR profile could still be obtained after running 10,000 steps on a dry surface. The researchers also identified the DNA profile of the footwear user [175].

Many studies researched the possibility of analyzing genetic material obtained from tissues exposed to water for different periods. Frippiat et al. [176] examined the influence of the aquatic environment on DNA persistence on blood stains on clothing, blood diluted with a sink’s drain water, and hair samples. The results showed that the STR analysis of DNA from blood stains on clothing immersed in canal water for a month is impossible: only one out of one hundred samples was able to determine 17 out of the 28 alleles analyzed. The DNA from blood diluted with the drainage water drain was significantly degraded after about 72 h; however, the results varied depending on the water sample used. After the same period, the genetic material of hair incubated in water also significantly degraded [176]. However, a full STR profile can be obtained for DNA isolated from blood stains deposited on the skin submerged in cold water if the analysis is performed within 48 h of the stain’s application. For comparison, it was possible to obtain a full STR profile for the analyzed contact DNA deposited on the leather material (after 30 s of strong hold), even when the immersion time was 7 days [177].

Very often, the perpetrator of a crime is trying to wash away biological traces [178]. Helmus et al. [179] researched how long it takes to rinse off epithelial cells from garments under running tap water. It turns out that a complete analysis can be performed, and a full DNA profile can be obtained for samples rinsed under running water for 10 min maximum. For comparison, the same results were obtained for the same samples soaked in a water bath, with or without soap, for one week. Therefore, it can be concluded that rinsing clothing under running water removes genetic material efficiently [179].

Often, forensic evidence is subjected to many environmental factors simultaneously [180]. It turned out that the DNA isolated from clothing placed in a pond in summer is only suitable for analysis for about 3.5 h from immersion, while in winter, this time is extended up to 2 weeks. Similarly, for samples placed in a river, it was 1 h in summer and 6 h in winter [179]. In another case, it was possible to obtain the genetic profile of a woman’s body found in a river with a slowly flowing current in which it stayed for 5.5 h. Additionally, STR analysis performed on the traces of saliva secured from a bite mark on the victim’s chest enabled to obtain the alleged perpetrator profile [181].

Similarly, it was possible to recover a full STR profile from fingerprints placed on an adhesive tape immersed in seawater for no longer than 1 week [182]. Much information on genetic material persistence in seawater was provided by Bertolini et al. [183], who analyzed the DNA isolated from the femurs and tibias of drowned bodies of immigrants excavated 15 months after a boat sinking in the Mediterranean Sea. In order to increase the probability of successful analysis, the forensic team used three different DNA extraction methods and multiple PCR amplifications using three different commercial kits. Thanks to this, it was possible to obtain reliable genetic profiles covering at least 16 out of the 21 analyzed STR markers in 70% of the samples [183]. It is worth mentioning that the bones used in the described study are characterized by a high probability of successful analysis [184].

For corpses immersed in water for a long time, it is worth isolating DNA from teeth, which are often well preserved. Kaur et al. [185] used this method in the case of a 40-year-old man found in a water reservoir a month and a half after disappearing. The remains were at the advanced decomposition stage, but DNA isolated from the tooth allowed to create and compare the victim’s STR profile with the profiles of relatives [185].

Water also significantly influences RNA’s degradation, as RNAase activity remains unchanged in water conditions, and dry conditions slow down the RNA degradation process [8,186]. MiRNA markers seem more stable than mRNA [187]. The degradation level of total RNA isolated from tissues has a negligible effect on the miRNA level measured in the tissue, while the level of mRNA decreases as total RNA degradation increases [188]. The extended half-life of miRNAs may result from the increased stability of molecules caused by the Argonaute proteins [189]. In the first 24 h after death, the level of miRNA markers remains constant or increases, while the mRNA level decreases in 2–24 h [60]. Mayes et al. [190] compared the stability of mRNA and miRNA exposed to changing environmental conditions (variable temperature and humidity). They found that mRNA could not be amplified after 30 days, while miRNA could for the entire duration of the experiment, i.e., 180 days [190]. Li et al. [191] showed that humidity and temperature significantly influenced mRNA and miRNA degradation. Complete mRNA profiles could be analyzed after 360 days in a dry environment; however, some markers disappeared after just 10 days in a humid environment [191].

Luminol and Bluestar Forensic are often used at crime scenes to visualize bloodstains [192,193]. Luminol works by chemiluminescence resulting from the oxidation of this compound, which is catalyzed by hemoglobin [194]. Bluestar Forensic is a newer luminol-based test with longer and more intense chemiluminescence [192]. Both Luminol and Bluestar Forensic do not degrade DNA in the revealed traces and enable subsequent STR analysis if the nucleic acid concentration in the sample is appropriate [195,196,197]. On the other hand, De Almeida et al. [198] showed that DNA analysis could not be performed 30 days after using Luminol and 120 days after using Bluestar Forensic on the sample [198]. However, the preparations for biological traces’ visualization often involve aqueous solutions, which dilute DNA in the traces, which, at a low initial concentration, may hinder the subsequent analysis of the genetic material [199].

In summary, water’s presence negatively affects the chances for successful analysis of genetic traces. Material stored in a humid environment degrades more than in a dry environment because of enhanced nucleic acid hydrolysis. Nevertheless, under the right conditions, it is possible to analyze contact DNA immersed in water, even if immersed for several days. Water temperature also affects the degradation rate. Since the aqueous solutions used for biological trace visualization may negatively affect the analysis of a small amount of genetic material, it is worth using such measures only when necessary.

### 3.6. Maceration

Maceration by removing soft tissues from the remains allows for assessing the skeleton for possible changes and injuries and obtaining a biological profile [200]. Contemporary maceration methods include thermal, bacterial, enzymatic, chemical, or invertebrate techniques [201]. Some of them use commercially available chemicals [202]. Enzymatic maceration generally appears less invasive than thermal maceration by boiling [203], while cold water maceration seems one of the safest methods for macerated tissues but is very time-consuming [204]. Maceration contributes to DNA degradation to a varying degree, irrespective of the applied maceration technique, which translates into the inability to analyze it [205,206], and sometimes it is necessary to isolate genetic material from macerated bone for later analyses [207].

Lee et al. [205] showed how various maceration techniques influenced the ability to amplify isolated nuclear DNA. Human amputated shanks were macerated using nine different techniques: hot water (approx. 90 °C), boiling water (100 °C), microwaving, bleaching (22 °C), hydrogen peroxide (3.5%, 22 °C), EDTA/papain (45 °C), meat softener (90 °C), sodium carbonate (90 °C), sodium carbonate with subsequent degreasing, and mechanical maceration as a control. The best results for DNA amplification were obtained for samples macerated with microwaves and sodium bicarbonate. Relatively good amplification results were obtained for samples macerated with EDTA/papain or bleach [205], even though Steadman et al. [208] showed greater loss of DNA after using the EDTA/papain maceration technique; however, in their case, the maceration lasted twice as long [208]. More importantly, Lee et al. [205] received positive results of DNA amplification after maceration by boiling [205]. This showed that high temperatures used for maceration do not always degrade the genetic material, despite previous studies reporting so [209]. Using meat softeners made the amplification only partially successful. The worst amplification coefficient was noted for samples macerated with hot water [205], most probably due to a too-long high-temperature exposure [208]. A very poor amplification was noted for samples macerated with hydrogen peroxide, and no amplification for samples macerated with sodium carbonate and degreasing agents was used simultaneously [205]. The authors proved that the maceration method affects the subsequent possibility of DNA analysis, and using microwaves and sodium bicarbonate enables successful subsequent DNA amplification. They concluded that the techniques requiring longer maceration times should be avoided [205]. Frank et al. [210] suggested that material for DNA analysis should be sampled before the maceration starts, if feasible [210].

In conclusion, maceration affects genetic material degradation. The safest process for nucleic acids is maceration using microwaves or sodium bicarbonate, and the worst effect came from hot water, with high temperatures causing additional damage. In practice, it is best to secure samples of genetic material for analysis before attempting maceration.

### 3.7. Radiation

The examination of biological material that precedes sampling DNA for further analyses often involves exposure to various types of radiation, such as UV- or X-radiation [211,212]. Hence, it is important to understand the influence of such radiation on the genetic material’s potential degradation.

Ionizing radiation can change and damage the DNA structure [213,214]. It was confirmed that X-rays negatively affect the amplification process of DNA isolated from X-ray-exposed bones [215], and even a single X-ray or CT examination of the biological material might decrease the amount of DNA that can be amplified after isolation [216]. Also, genetic material mutates after UV radiation exposure [217]. Formed pyrimidine dimers, the most frequently observed UV-induced lesions in living tissues [218], distort DNA strands, which may inhibit the replication and transcription processes [219,220]. Exposure to UV-C radiation for over 100 h caused single-strand breaks in DNA isolated from dry bloodstains, which was the main observed damage, along with double-strand breaks and pyrimidine dimerization, and made STR profile analysis impossible [221]. Biological traces are less prone to DNA damage caused by UV-A and UV-B reaching the Earth’s surface. The genetic material enclosed in the cells in bloodstains is protected from degradation that interferes with STR analyses. However, in the case of dried blood spots, reactive Fe^2+^ cations released from hemoglobin might add to the DNA damage [222]. Acellular DNA is more susceptible to radiation, especially as the humidity of the environment increases, and thread breaks are more frequent [223].

In conclusion, both X-ray and UV radiation negatively affect the genetic material’s integrity, and cell-free DNA is most sensitive to radiation. The successful DNA analysis depends on the sample’s minimal exposure to any radiation.

The results of studies on the influence of various factors on the persistence of nucleic acids are presented in Table 1.

## 4. Future Directions

The STR analysis is a method of identifying people that is used worldwide. Future methods of analyzing genetic material for forensic medicine purposes should be expected to become faster and more sensitive and should constitute more valuable evidence [224]. Rapid and automated DNA testing will undoubtedly attract attention in the future. The new instruments combine multiple steps: DNA extraction, rapid PCR amplification, DNA separation, detection, size determination, and genotyping. The use of these single-instrument profiling platforms to generate DNA profiles is called Rapid DNA. Such systems shorten the amplification time to several minutes [225]. The analysis time will become shorter until the limit for this method is reached, which may be the polymerase operation time or the primer annealing time. This would enable genetic trace analysis immediately at the crime scene or during a short-term arrest of a suspect. Rapid DNA has already been used to identify victims at crash sites. A real-time connection with databases will be necessary to exploit their full potential [225,226]. Recent cases of using databases showed that it is possible to identify the murderer and time. Analysis of scrapings from the victims’ fingernails yielded an STR profile that was initially not listed in any database. Five years later, the same individual broke into a church and injured their hand on broken glass. Blood trace analysis and a database search resulted in a positive match with a database DNA profile connected to a double homicide. Database searches and DNA profile matching enabled the long-due conviction [227]. Next-generation sequencing (NGS) technologies will play an increasingly important role [228]. They will overtake the limitations of the most commonly used technologies. NGS enables increased discriminatory power for STR analysis and provides new possibilities for human identification [229]. Base-by-base sequencing detects variants in the repeat and flanking region, enhancing the discrimination power [230]. Despite many studies showing NGS’s usefulness in forensics, it is still not implemented in many forensic laboratories, most probably due to its high labor intensity and high costs per sample [231]. Single-cell sequencing might be more widely used [232]. This technique reveals the unique gene expression of each cell type, providing directions for exploring cell heterogeneity, cell type-specific responses to injury or disease, and the mechanisms underlying these processes. It can also be used in forensic medicine [233]. Sigle-cell sequencing can be a promising tool for use to deconvolute mixed traces submitted for forensic DNA phenotyping [234]. Also, the use of artificial intelligence in forensic medicine has been talked about [235]. The development of environmental DNA analysis will probably play a significant role in the future, which will significantly help determine the origin of the tested samples [236]. Finally, the development of CRISPR technology will also be significant for forensic medicine [237].

In conclusion, forensic genetics is a rapidly developing field. The methods described in this review will improve in the coming years, and new ones will certainly be developed. It may be necessary to introduce large international databases of DNA profiles, and it seems imperative to follow the latest reports in this field. However, eligible material is necessary for every genetic analysis. The description of the nucleic acid degradation factors presented in this review may help choose suitable preservation methods, which may increase the chances for full analysis, regardless of the method of choice.

## 5. Summary

Biological traces containing genetic material are often irrefutable evidence that allows the perpetrator of a crime to be convicted or the suspect acquitted. It is not always possible to analyze the genetic material due to the influence of many factors causing partial or complete degradation of DNA and RNA present in sampled tissues or biological traces. In this review, we have covered the basics of genetic profiling. In one place, we have gathered studies reporting on various factors influencing nucleic acid degradation. Temperature, water environments, time, radiation, maceration, cleaning agents, and pHs were taken into consideration. Based on the available studies, it can be concluded that an increase in humidity leads to an increase in the genetic material’s degradation rate by accelerating its hydrolysis. Also, high temperature adversely affects genetic material persistence, while a low temperature is suitable for the long-term storage of genetic material samples. The available data on the persistence of DNA and RNA after attempts to remove them with chemicals, such as washing with detergents, washing in a washing machine, dishwasher, or bath, and the influence of environmental pHs, were analyzed. It has been shown that biological traces can often be successfully analyzed, despite the previous use of cleaning agents. Particularly noteworthy is the persistence of sperm on laundered fabrics and the possibility of their transfer to other materials during washing. Examples of possible analyses of ancient samples dating back several thousand years were presented, as well as the possibility of obtaining human DNA from the digestive tracts of larvae and excrements of flying insects that feed on carcasses. The possibility of analyzing contact DNA from various subjects was also presented. More importantly, the review discusses the influence of stains used by forensic scientists for biological trace visualization, various types of radiation, or maceration methods used during the forensic examination of DNA degradation. The work repeatedly refers to criminal cases resolved thanks to the analysis of recovered genetic material. The analyzed studies allow us to conclude that nucleic acid degradation is affected by many factors acting at the same time to varying degrees, which makes comparing the conditions and predicting the nucleic acid degradation rate difficult. Whenever possible, the sample for genetic testing should be collected before any other activity adversely affects the success of the analysis. Finally, we outlined future directions in forensic genetics and mentioned methods that may soon be used on a large scale or be modernized.

## Figures and Tables

**Table 1:** Characteristics of various factors: water environment, temperature, pH, cleaning agents, preparations for biological traces’ visualization, maceration, and radiation influencing the persistence of nucleic acids.

Analyzed Material and Source	Conditions of the Experiment	Duration	Results of Genetic Material Analysis	Reference
Water environent				
DNA from the blood stain on the shoe sole	Walk on a dry surface, 10,000 steps	-	Partial degradation, full STR analysis possible	Schmidt et al., 2022 [175]
	Walk on a wet surface, 10 steps		Almost complete degradation	
DNA from blood stains	Blood-stained clothing placed in the water channel	1 month	Complete degradation	Frippiat et al., 2017 [176]
DNA from water-diluted blood	Blood diluted with sink drain water	72 h	Complete degradation	
Hair DNA	Hair submerged in water	72 h	Complete degradation	
DNA from blood stains on the skin	Blood-spotted skin submerged in cold water	48 h	Partial degradation, full STR analysis possible	Meixner et al., 2020 [177]
Touch DNA sample	Skin submerged in cold water	7 days	Partial degradation, full STR analysis possible	
DNA from epithelial cells on clothing	Clothes rinsed under running water	10 min	Partial degradation, full STR analysis possible	Helmus et al., 2018 [179]
	Clothes placed in a bathtub with soapy water	7 days	Partial degradation, full STR analysis possible	
	Clothes immersed in the pond in summer	3.5 h	Partial degradation, full STR analysis possible	
	Clothes immersed in the pond in winter	2 weeks	Partial degradation, full STR analysis possible	
	Clothes dipped in the river in summer	1 h	Partial degradation, full STR analysis possible	
	Clothes dipped in the river in winter	6 h	Partial degradation, full STR analysis possible	
Contact DNA from duct tape fingerprints	Duct tape immersed in seawater	7 days	Partial degradation, full STR analysis possible	Forger et al., 2021 [182]
Temperature				
DNA from blood samples	Samples incubated at 55 °C and 41% humidity	11 days	Complete degradation	Abdulla et al., 2021 [122]
	Samples incubated at 35 °C and 55% humidity	21 days	Partial degradation, full STR analysis possible	
	Samples incubated at 25 °C and 58% humidity	21 days	Partial degradation, full STR analysis possible	
	Samples incubated at 4 °C and 68% humidity	21 days	No degradation detected	
mtDNA from skeletal remains	Bones burnt at <600 °C	-	Complete reconstruction of the mtDNA genome possible	Emery et al., 2022 [127]
DNA from passive blood stains	Stains stored at −20 °C, 20 °C, and 40 °C	15 days	Relationship between higher temperature and increased DNA degradation rate confirmed	Cossette et al., 2021 [119]
RNA from blood stains	Stains incubated at different temperatures and relative humidity	-	RNA degradation rate decreases 5–10× while decreasing from 37 °C to 20 °C or from 75% to 35% relative humidity	Heneghan et al., 2021 [124]
pH				
DNA from skeletal remains	Two bodies attempted to be dissolved in concentrated HCl and H_2_SO_4_ mixture	21 days	High degree degradation, DNA identification of only one victim possible	Vermeij et al., 2015 [135]
Teeth	Teeth incubated in 25 mL 37% HCl	15 h	Material dissolved completely	Jadhav et al., 2009 [140]
	Teeth incubated in 25 mL 65% HNO_3_	20 h	Material dissolved completely	
	Teeth incubated in 25 mL 96% H_2_SO_4_	6 days	Residual sludge at the bottom of the container	
DNA from saliva and oral mucosa	Samples obtained from the bite marks on a fruit	21 days	Partial degradation, full STR analysis possible	Pfeifer et al., 2017 [48]
Nail samples	Samples incubated in 50% NaOH	7 days	Material dissolved completely	Tran & Jasra, 2020 [141]
Cleaning agents				
DNA from sperm stains	Fabric with stains machine-washed 6×	-	Partial degradation, full STR analysis possible	Noël et al., 2017 [152]
DNA from saliva stains	Fabric with stains machine-washed 3×			
DNA from semen stains	Fabric with 8-month-old stains several times machine-washed with detergent at 60 °C	-	Partial degradation, full STR analysis possible	Brayley-Morris et al., 2015 [153]
DNA from blood stains	Fabric with stains machine-washed at 40 °C	-	Partial degradation, full STR analysis possible	Kulstein & Wiegand 2018, [172]
	Fabric with stains machine-washed at 60 °C			
DNA from blood stains	Fabric with stains machine-washed at 90 °C	-	Partial degradation, full STR analysis possible	Alice et al., 2016 [151]
DNA from blood stains	Fabric with stains machine-washed at 90 °C	-	Complete degradation	Ünsal et al., 2021 [161]
mRNA	Samples exposed to changing temperature and humidity	30 days	Amplification possible	Mayes et al., 2019 [190]
mRNA	Samples exposed to changing temperature and humidity	180 days	Amplification not possible	
mRNA	Samples incubated in a dry environment	360 days	No degradation	Li et al., 2021 [191]
	Samples incubated in a humid environment	10 days	Partial degradation	
DNA from saliva deposited on the victim’s body	Samples exposed to shower water	-	Y chromosome STR analysis possible: complete profile obtained for majority of samples	Williams et al., 2015 [164]
DNA from vaginal lavage	Vaginal lavage performed with delay	100 h	Full STR analysis possible	Naresh et al. 2017 [99]
DNA from blood traces, epithelial cells, and saliva located on knife	Knife rinsed with water	-	Partial degradation, full STR analysis possible	Helmus et al., 2020 [166]
	Knife washed by hand with detergent			
	Knife washed in a dishwasher		Complete degradation	
Preparations for biological traces visualization				
DNA from biological traces	Materials treated with Luminol or Bluestar Forensic + immediate DNA analysis	-	Full STR analysis possible	Manna & Montpetit, 2000 [195]; Jakovich, 2007 [196]; Tobe et al., 2007 [197]
DNA from biological traces	Material treated with Luminol + delayed DNA analysis	30 days	Complete degradation	Almeida & Glesse, 2011 [198]
	Material treated with Bluestar Forensic + delayed DNA analysis	120 days		
Maceration				
DNA from macerated bones	Maceration by microwaving	-	Amplification possible	Lee et al., 2010 [205]
	Maceration with NaHCO_3_ at 90 °C			
	Maceration with EDTA/papain at 45 °C			
	Maceration with bleach at 22 °C			
	Maceration by boiling			
	Maceration with water at 90 °C		Almost complete degradation	
	Maceration with 3.5% H_2_O_2_ at 22 °C			
Radiation				
DNA from X-rayed bones	Bones after one X-ray examination	-	Decreased amount of amplifiable DNA	Grieshaber et al., 2008 [216]
	Bones after one CT scan			
DNA from cells present in bloodstains	Stains exposed to UV-C radiation	100 days	Complete degradation	Hall & Ballantyne, 2004 [221]
	Stains treated with UV-A radiation		Full STR analysis possible	
	Stains treated with UV-B radiation			

## Data Availability

Not applicable.
